# Correction for Deaven et al., “*Salmonella* Genomics and Population Analyses Reveal High Inter- and Intraserovar Diversity in Freshwater”

**DOI:** 10.1128/aem.01227-25

**Published:** 2025-09-18

**Authors:** Abigail M. Deaven, Christina M. Ferreira, Elizabeth A. Reed, Jeremy R. Chen See, Nora A. Lee, Eduardo Almaraz, Paula C. Rios, Jacob G. Marogi, Regina Lamendella, Jie Zheng, Rebecca L. Bell, Nikki W. Shariat

## AUTHOR CORRECTION

Volume 87, no. 6, e02594-20, 2021, https://doi.org/10.1128/aem.02594-20. Page 12: The following should be added to Materials and Methods.

“Figure 3 was generated using SankeyMatic (http://sankeymatic.com/build/). The Sankey plots were exported as high-resolution PNG files, and the labels (sample ID on the left, and serovar name on the right) were added in PowerPoint. These files were saved as PDFs for submission.”

The authors would like to apologize for any confusion caused.

